# Fractal analysis of plaque border, a novel method for the quantification of atherosclerotic plaque contour irregularity, is associated with pro-atherogenic plasma lipid profile in subjects with non-obstructive carotid stenoses

**DOI:** 10.1371/journal.pone.0192600

**Published:** 2018-02-12

**Authors:** Francesco Moroni, Marco Magnoni, Vittoria Vergani, Enrico Ammirati, Paolo G. Camici

**Affiliations:** 1 Cardiothoracic and Vascular Department, Vita-Salute University and San Raffaele Hospital, Milan, Italy; 2 De Gasperis Cardio Center, Niguarda Ca’ Granda Hospital, Milan, Italy; Universidad Francisco de Vitoria, SPAIN

## Abstract

**Background and aims:**

Plaque border irregularity is a known imaging characteristic of vulnerable plaques, but its evaluation heavily relies on subjective evaluation and operator expertise. Aim of the present work is to propose a novel fractal-analysis based method for the quantification of atherosclerotic plaque border irregularity and assess its relation with cardiovascular risk factors.

**Methods and results:**

Forty-two asymptomatic subjects with carotid stenosis underwent ultrasound evaluation and assessment of cardiovascular risk factors. Total, low-density lipoprotein (LDL), high-density lipoprotein (HDL) plasma cholesterol and triglycerides concentrations were measured for each subject. Fractal analysis was performed in all the carotid segments affected by atherosclerosis, i.e. 147 segments. The resulting fractal dimension (FD) is a measure of irregularity of plaque profile on long axis view of the plaque. FD in the severest stenosis (main plaque FD,mFD) was 1.136±0.039. Average FD per patient (global FD,gFD) was 1.145±0.039. FD was independent of other plaque characteristics. mFD significantly correlated with plasma HDL (r = -0.367,p = 0.02) and triglycerides-to-HDL ratio (r = 0.480,p = 0.002).

**Conclusions:**

Fractal analysis is a novel, readily available, reproducible and inexpensive technique for the quantitative measurement of plaque irregularity. The correlation between low HDL levels and plaque FD suggests a role for HDL in the acquisition of morphologic features of plaque instability. Further studies are needed to validate the prognostic value of fractal analysis in carotid plaques evaluation.

## Introduction

Atherosclerosis of the carotid arteries is a widely recognised risk factor for ischemic stroke. It is estimated that one in five ischemic strokes originates from carotid plaques, mainly due to an arterio-arterial embolization.[1, 2] Among carotid plaque characteristics of prognostic value, plaque irregularity appears to have a promising role in the identification of subjects at risk.[3–5] However, most of the studies evaluating plaque irregularity rely on qualitative classification schemes lacking in standardization, which led to inconsistent results in a recently published meta-analysis.[6] The development of quantitative methods for the evaluation of plaque irregularity may offer the advantage of higher reproducibility, eventually leading to more robust results. Fractal analysis provide a method for quantifying the complexity of biological and anatomical structures.[7, 8] The measure obtained is the fractal dimension (FD), a unitless number that expresses how the analyzed structure fills the space: the higher the FD, the more complex and irregular is the object of interest.[8, 9] The range of FD is determined by the topology of the structure: similarly to that of a coastline, the FD of an atherosclerotic plaque border is expected to fall between 1 and 2.[8] In the present study, we propose a novel fractal analysis-based method for the quantification of carotid artery plaque irregularity. In addition, we explore the relation between plaque irregularity and other ultrasonographic plaque characteristics and cardiovascular (CV) risk factors.

## Materials and methods

### Study population

The population included in this study represent a subpopulation of a larger prospective study (Imaging della PLAcca Carotidea, IMPLAC) aimed at identifying carotid plaque features associated with subclinical cerebral damage.[10]. The subjects were consecutively enrolled among patients referred to Ospedale San Raffaele Vascular Surgery outpatient service for carotid ultrasound evaluation. Inclusion criteria were: i) absence of symptoms for cerebrovascular disease and ii) carotid stenosis with no current indication for revascularization, i.e. determining a reduction in diameter between 40% and 70%. Among the IMPLAC population, only subjects with high quality B-mode long-axis image of the carotid plaque were enrolled/considered in the current study, yielding a total of 42 subjects.

The study received the approval of the of San Raffaele Institute Ethics Committee (date of approval January, 30^th^ 2012, protocol name IMPLAC, protocol approval number IMPLAC11006333). All participants provided written informed consent.

### Clinical evaluation

Upon enrolment in the study, each patient underwent a thorough clinical examination aimed at identifying CV risk factors. Ten-year general CV risk was evaluated using Framingham risk score. Subjects were considered at high risk if estimated CV risk exceeded 20% at 10 years, were diabetic or had a personal history of symptomatic CV disease.[11] A 5-ml peripheral blood sample was collected with EDTA-anticoagulated vacutainer tubes on the same day of the ultrasonographic evaluation. Glycaemia, total cholesterol, high-density lipoprotein cholesterol (HDL-C), low-density lipoprotein cholesterol (LDL-C), Apolipoprotein A-I (ApoA-I), Apolipoprotein B (ApoB) and high-sensitivity C-reactive protein (hsCRP) were measured using a colorimetric method using the Cobas Mira Plus analyzer (Horiba, ABX, France), as previously described.[10] Creatinine level taken within 3 months from enrollment, if available, was recorded as part of past medical history. Glomerular filtration rate was estimated using Cockroft-Gault formula.

### Carotid artery ultrasound

All patients underwent bilateral carotid examination by the same operator (MM) using dedicated ultrasonography equipment (Logiq S8, GE Healthcare, Little Chalfort, UK) and 7-MHz linear probe (7L, GE Healthcare, Little Chalfort, UK). The operator was blinded to clinical and biochemical data. The degree of stenosis was estimated from Doppler velocities according to the Society of Radiologists in Ultrasound Consensus Conference.[12] Two independent examiners (FM and MM) visually categorized the plaques as irregular or regular and identified, for each patient, the plaque with the highest degree of stenosis, hereon referred to as main plaque. Any discordance was resolved by consensus. The main plaques were subsequently characterized according to their echogenicity in one of five classes of progressively increasing greyscale as previously described.[13] For subsequent analysis, plaques echogenicity was dichotomized as follows: classes I-III were considered lipid-rich while plaques in classes IV-V were considered fibrocalcific. Common carotid intima-media thickness (CC-IMT) was measured on the far wall 10mm proximal from the bifurcation as previously described.[14]

### Fractal analysis of carotid plaque border

**Fig 1:**
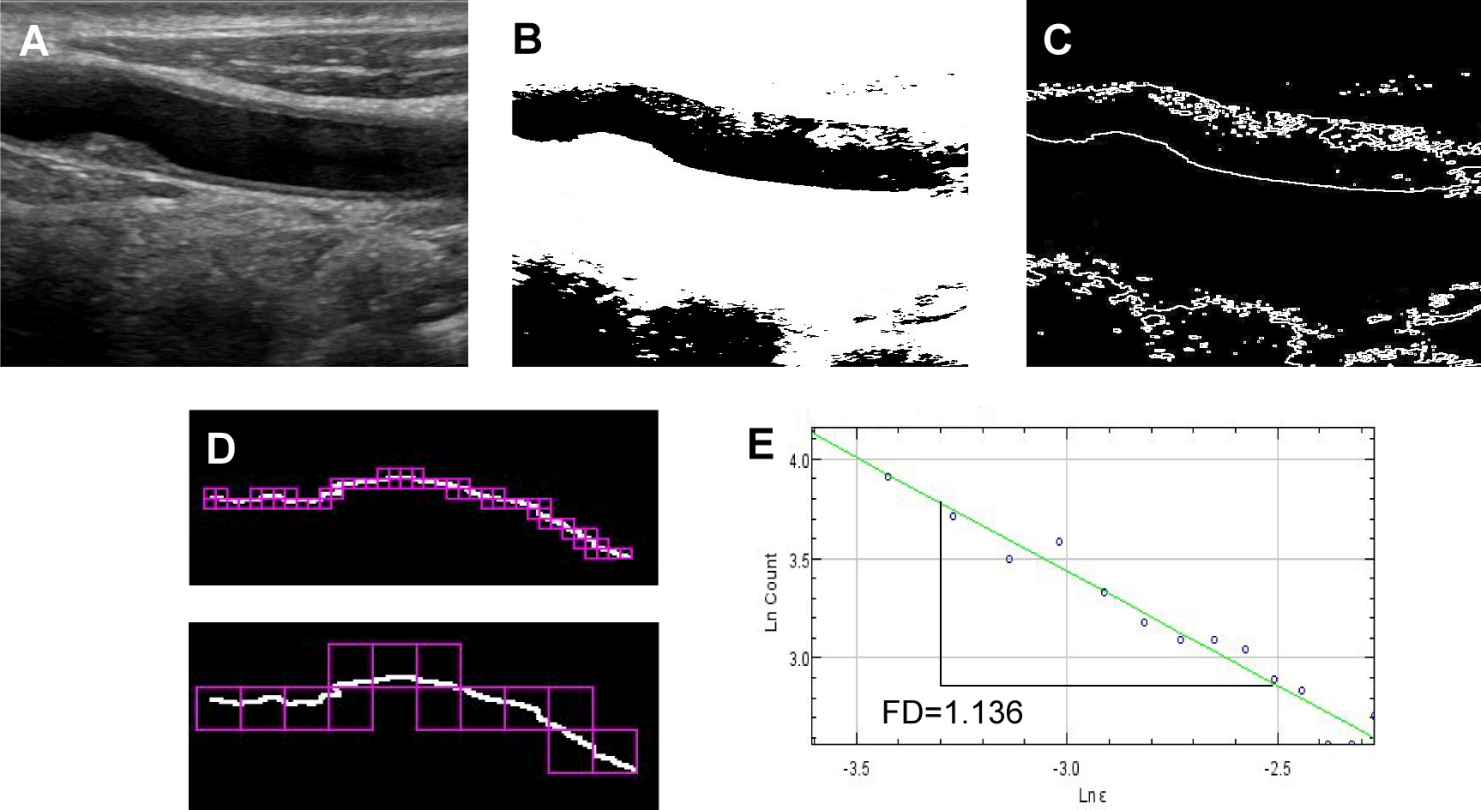
Fractal analysis of a plaque located in the right carotid artery of a representative subject. Panel A shows the longitudinal view of a non-obstructive plaque located in the right common carotid artery. Panel B shows the same image after binarization using a modified IsoData algorithm, while Panel C displays contour extraction by computing edges in the areas of highest gradient magnitude using the Sobel operator. Panel D shows two grids of different scales generated as part of the evaluation of fractal dimension (FD) using a box counting algorithm. The FD of the plaque border corresponds to the opposite of the slope of logarithmic plot of the number of boxes containing the objects (Y, Ln Count) vs. the dimension of the boxes side (X, Ln**ε**). The higher is the FD, the higher is the irregularity of the plaque surface.

**Fig 2:**
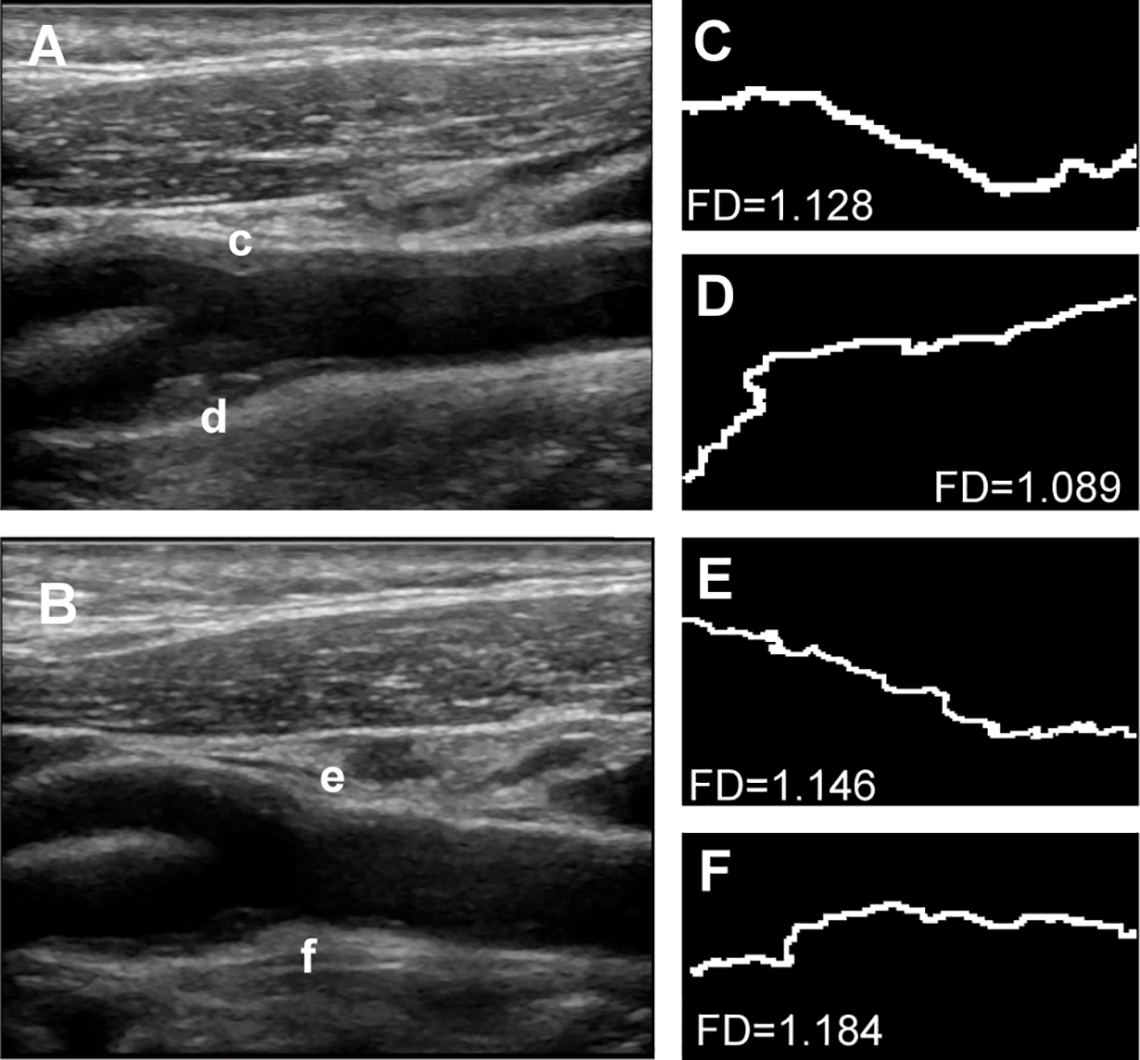
Fractal analysis of all segments involved by atherosclerosis in a representative subject. Panel A shows the image of the right carotid bifurcation, in which segments affected by atherosclerosis can be identified: the internal carotid artery (denoted with c) and the carotid bulb, the latter by a plaque extending in the external carotid artery (d). Panel B displays the left carotid bifurcation of the same patient. Again, two involved segments can be identified: the internal carotid artery (by a plaque extending from the carotid bulb, e) and the carotid bulb (f). Panels C-F display the magnified contour extracted from plaque c-f respectively. Fractal dimension (FD) for each contour is indicated in each panel. The total number of involved segments in this patient was 4. Average FD was 1.137.

Fractal analysis was performed on long axis views of carotid artery plaques using the open source image processing software Fiji (Fiji Is Just ImageJ, https://fiji.sc/).[15] First, the images were thresholded and binarized using an automated thresholding technique, a modified variation of the IsoData algorithm, default thresholding method in Fiji, as previously described.[9] Plaque border was extracted by computing edges in the areas of highest gradient magnitude using the Sobel operator.[16] Fractal analysis was then performed using Frac-Lac Fiji plug-in (Karperien, A., v2.5) using the box counting method and default settings as previously described.[8, 17] FD was measured for all plaques in common carotid, carotid bulb and internal carotid artery. For each patient, we recorded the FD of main plaques (main plaque FD, mFD), and the average FD of all identifiable plaques (global FD, gFD). Fig 1 shows the image processing for fractal analysis in the main plaque of a representative subject, while Fig 2 depicts the analysis of gFD in a representative subject.

### Statistical analysis

All continuous variables were tested for normality using Shapiro-Wilk normality test and are expressed as mean ± standard deviation (SD) or median [interquartile range (Q1-Q3)] as appropriate. Categorical variables are summarized as absolute frequency (percentage). Group differences were tested using Student’s t-test or Mann-Whitney U test as appropriate. Spearman’s rank correlation coefficient was used to assess statistical dependence. Multivariate regression analyses were performed using a general linear model to identify independent associations between FD and plaque characteristics and CV risk factors. All variables significantly associated with FD were included in the model. Inter-observer reproducibility of fractal analysis was assessed on a subset of 20 main plaques reviewed by independent operators. Intra-observer reproducibility was assessed on the same subset of 20 subjects, reviewed in a random order after 1 month from the first analysis. Paired FD measures were analyzed with Bland-Altman method and intraclass correlation coefficients (ICC), as well as by calculating repeatability and variation coefficients. Group differences or correlations with p<0.05 were considered significant. All statistical analysis were performed using GraphPad Prism 5 (GraphPad Software Inc., La Jolla, USA) or R v3.1.2.

## Results

### Study population

Forty-two subjects were included in the final population of the study. Mean age was 70±8 years and 24, 57%, were males. Eighty-one percent of subjects suffered from hypertension and 76% had a clinical history of dyslipidemia. Nineteen percent of subjects suffered from diabetes mellitus type 2 and 14% had a clinical history of symptomatic coronary artery disease. Median Framingham risk score was 10% at 10 years. Twenty subjects, 48%, fulfilled our definition of high CV risk. Table 1 summarizes population clinical characteristics and blood tests results.

**Table 1:** Population characteristics.

	Patients (n = 42)
**Demographic Characteristics**	
Age, years	70±8
Male, n (%)	24 (57)
**Cardiovascular Risk Factors**	
Family history of coronary artery disease, n(%)	12 (29)
Family history of stroke, n(%)	5 (12)
Systemic arterial hypertension, n(%)	34 (81)
Resistant hypertension, n(%)	7 (17)
Hypercholesterolemia, n(%)	32 (76)
Type 2 diabetes mellitus, n(%)	8 (19)
Current smoker, n(%)	6 (14)
Previous smoker, n(%)	20 (48)
Body mass index (kg/cm^2^)	25±4
Framingham risk score (%)	10 (5–20)
High cardiovascular risk n(%)	20 (48)
**Cardiovascular History**	
History of CAD	6 (14)
Previous acute coronary syndrome, n (%)	4 (10)
**Clinical Biochemistry**	
Total cholesterol (mg/dL)	175±32
LDL cholesterol (mg/dL)	103±26
HDL cholesterol (mg/dL)	41 (36–47)
Triglycerides (mg/dL)	133 (99–164)
Triglycerides-to-HDL	3.3 (2.0–4.1)
ApoA1 (mg/dL)	147±27
ApoB (mg/dL)	73 (62–86)
Glycaemia (mg/dL)	97 (90–124)
hsCRP (mg/L)	1.13 (0.55–2.8)
eGFR (mL/min)	72±21
**Current Treatment**	
ACEi/ARB, n (%)	30 (71)
Statins, n (%)	24 (57)
β-blockers,n (%)	14 (33)
Antiplatelet medications, n (%)	28 (67)
**Carotid Plaque Ultrasound**	
Stenosis (Doppler), n (%)	
<50%	24 (57)
50–70%	18 (43)
CC-IMT	0.82 (0.75–1.23)
Irregular plaques, n (%)	25 (60)
Lipid-rich plaques, n (%)	12 (29)
Fibrocalcific plaques, n (%)	30 (71)
Main plaque fractal dimension	1.136±0.039
Global fractal dimension	1.145±0.039

CAD = coronary artery disease; LDL = low density lipoprotein, HDL = high density lipoprotein, hsCRP = high sensitivity C-reactive protein, eGFR = estimated glomerular filtration rate, ACEi = Angiotensin converting enzyme inhibitors, ARB = angiotensin receptor blockers.

### Carotid artery ultrasound

A total of 147 carotid artery segments (common carotid, carotid bulb or internal carotid artery) out of 252 examined were involved by atherosclerosis. Four subjects had at least one plaque with significant calcifications. Plaque border of these plaques could not be entirely visualized, thus assessment of echogenicity and evaluation of gFD was not possible. No main plaque presented significant calcification impairing image quality and analysis. The median number of involved segments per patient was 4. Eighteen subjects, 43% of total, had a main plaque stenosis of 50–70% in terms of diameter according to Doppler criteria, while the remaining 24 had a stenosis of less than 50% in diameter. Thirty subjects, 71%, had a fibrocalcific (class IV-V) main plaque according to plaque echogenicity. Twenty-five subjects (60%) had a main plaque which was visually categorized as irregular. CC-IMT was 0.82 (0.75–1.23). Table 1 summarizes carotid artery ultrasound parameters.

### Fractal analysis of plaque border

**Fig 3:**
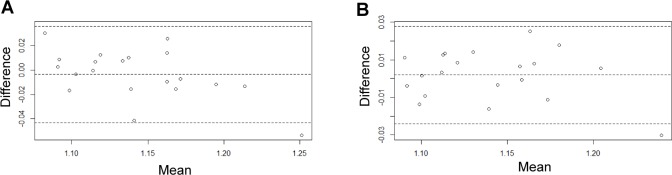
Bland Altman plot for fractal analysis. Panel A shows Bland-Altman plot for inter-operator reproducibility analysis, while Panel B shows results for intra-operator analysis.

No subject had significant calcification with posterior acoustic shadow within the main plaque, thus plaque border could be entirely visualized in all images. Mean mFD was 1.136±0.039. On the other hand, 4 subjects had at least one plaque with significant calcifications, in which gFD could not be evaluated. In the 38 remaining patients, average gFD was 1.145±0.039. Table 1 displays FD values. Fractal analysis showed a good inter- and intra-observer reproducibility, with no bias evident at Bland Altman analysis (Fig 3, Panels A and B respectively). ICC showed a satisfactory correlation between repeated measures both in the inter-observer (0.9, 95% confidence interval 0.77–0.96) and intra-observer (0.95, 95% confidence interval 0.87–0.98) evaluations. Repeatability coefficients were 0.040 and 0.026 for inter-observer and intra-observer assessments, respectively. Coefficients of variation were less than 2% for both analyses.

### Plaque border FD and other known plaque risk features

mFD was not associated with either the degree of stenosis (1.132±0.041 in subjects with a stenosis <50% and 1.141±0.037 in subjects with a stenosis of 50–70% in diameter, p = 0.44) or plaque echogenicity (1.127±0.49 in lipid-rich plaques vs 1.134±0.034 in fibrocalcific plaques, p = 0.36). Plaque that were visually categorized as irregular had a higher mFD when compared to those considered regular, but the difference did not reach statistical significance (1.143±0.038 vs 1.124±0.039, p = 0.11).

### Plaque border FD and CV risk factors

**Fig 4:**
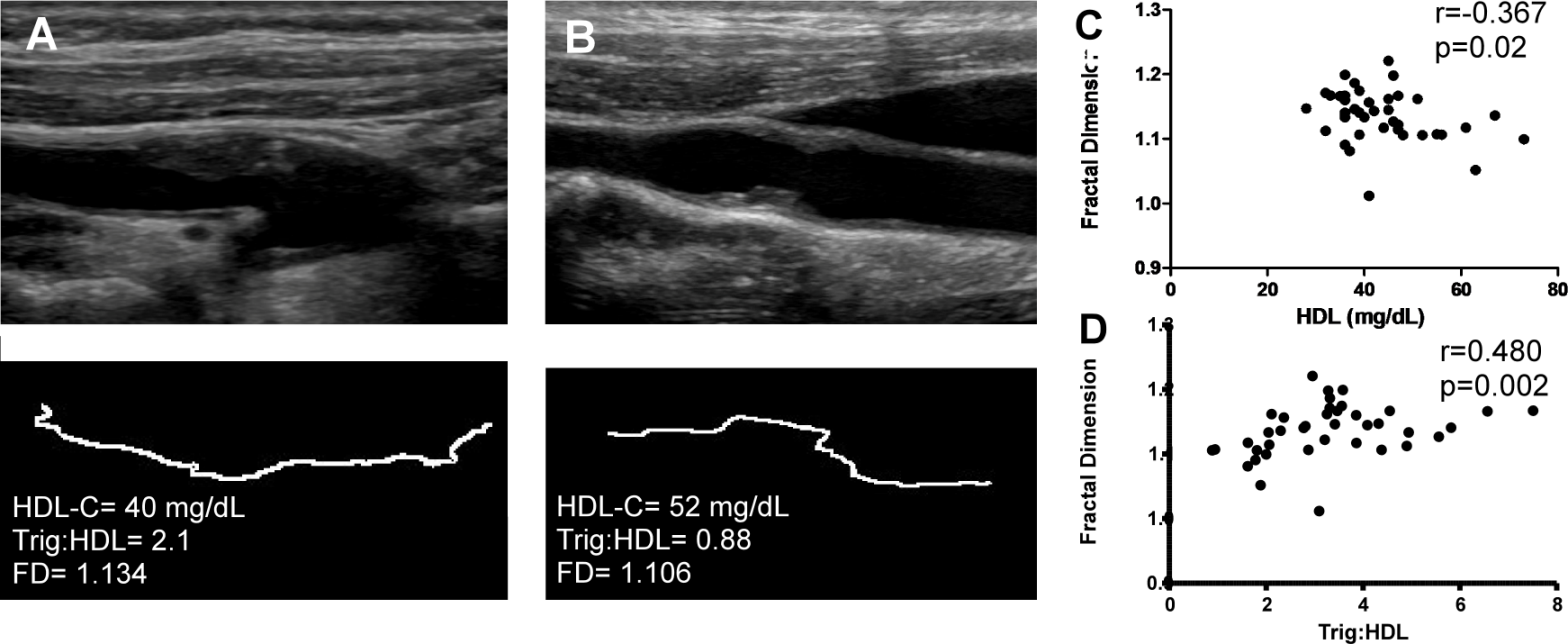
Correlation between fractal dimension, plasma HDL cholesterol and triglycerides-to-HDL ratio. Panel A-B compare and contrast two representative patients. The subject in Panel A appear to have a higher plaque border complexity and lower HDL-C compared to the subject in panel B. Lower panels show a magnified image of plaque contour. Panel C displays the scatter plot of fractal dimension versus HDL-C, while Panel D shows the scatter plot of fractal dimension versus triglycerides-to-HDL ratio. HDL-C = high density lipoprotein cholesterol; FD = fractal dimension; Trig:HDL = triglycerides-to-HDL ratio.

No sex difference in both mFD and gFD was evident in the study population. Age showed a borderline significant positive correlation with mFD (r = 0.245, p = 0.07) and no correlation with gFD (r = -0.169, p = 0.31). No significant association was found between mFD or gFD and diabetes, hypertension, past history of CAD or smoking status. Neither ten-year Framingham risk score (r = 0.223, p = 0.17 for mFD, r = -0.083, p = 0.629 for gFD), nor high CV risk status (mFD 1.136 for high risk vs 1.135 for non-high risk, p = 0.92 and gFD 1.140 for low risk vs 1.150 for high risk, p = 0.70) were significantly associated with FD. No significant correlation was found between blood levels of hsCRP and mFD or gFD. Total cholesterol plasma levels did not correlate with either mFD or gFD, as was the case for LDL-C. Interestingly, mFD displayed a significant inverse correlation with plasma HDL-C (r = -0.367, p = 0.02) and a trend towards significant correlation with blood triglycerides levels (r = 0.284, p = 0.07). Furthermore, the plasma triglycerides-to-HDL ratio, a known marker for metabolic syndrome and highly atherogenic dyslipidemia,[18] showed a highly significant correlation with mFD (r = 0.480, p = 0.002). Since the association between lipid profile and mFD may be influenced by current lipid lowering therapy, we performed a sensitivity analysis regarding current statin use. No significant difference in terms of mFD was found between subjects taking or not taking statins (1.145 vs 1.135, p = 0.77). On multivariable analysis, plasma HDL-C and triglycerides-to-HDL ratio remained significantly associated to mFD after adjusting for statin use (p = 0.05 and p = 0.03 respectively). However, ApoA1 and ApoB levels were not significantly correlated with either mFD or gFD. Fig 4 displays the relation between mFD, plasma HDL-C and triglycerides-to-HDL ratio.

## Discussion

In this proof of principle study, we described the use of fractal analysis in the quantification of carotid plaque irregularity and found an inverse correlation between mFD, HDL-C plasma levels and triglycerides-to-HDL ratio in an asymptomatic population, independently of current statin treatment. Of note, mFD and gFD were not associated with other carotid plaque characteristics and other CV risk factors.

Fractal analysis is a sophisticated mathematical tool that allows quantitative assessment of the complexity of an object or phenomenon. The use of FD to describe biological structures and physiological processes has been proposed, and has shown promising results, in many medical fields: among others, it has various applications in neurology,[19] oncology[20] and CV sciences.[8] This study shows the applicability of fractal analysis also in the study of carotid plaques and, in particular, of their profile on long axis view images. Previous literature mainly relied on a qualitative dichotomous classification (smooth vs. irregular) of plaque profile.[3, 4] Of the few reports proposing quantitative evaluation of plaque irregularity, most relied on the spatial and tissue characterization provided by advanced imaging modalities, including magnetic resonance imaging or 3D ultrasound, [21, 22] which will unlikely be available for routine clinical practice in the near future. To the best of our knowledge, only two previous studies assessed plaque irregularity quantification on standard ultrasound images, both of which involved the quantification of plaque border curvature.[23, 24] Both the techniques employed in-house developed software, which limit their diffusion, and have limited validation in terms of reproducibility.[23, 24]. On the other hand, the Fiji software used for fractal analysis is open source. Moreover, differently from the just mentioned techniques, fractal analysis provides a measure of the amount of complexity, and thus may aid in the definition of finer morphological description of the plaque and, possibly, in a better definition of the risk.

The analysis of the relation between FD and CV risk factors shows a greater FD in the context of a deregulated lipid profile, thus conferring a biological value to fractal analysis. We see an inverse correlation between HDL-C and plaque FD. This finding is in line with previous evidence of associations between low levels of plasma HDL-C and morphologic features of high risk carotid plaques.[25, 26] The reason for the increased plaque irregularity in conditions of reduced plasma HDL-C remains currently unknown, but lends itself to speculation. Low circulating HDL-C, and the resulting reduced cholesterol export from plaque macrophages is known to lead to an accumulation of plaque foam cell,[27] which was shown to directly correlate to surface irregularity in animal models of atherosclerosis.[28] Furthermore, subjects with low plasma HDL-C levels were shown to have higher matrix-metalloproteinase activity,[29] which are involved in the process of plaque destabilization, including erosion and rupture.[30, 31] On the other hand, it is reasonable to think that plaques with vulnerable characteristics, such as ulcers, thrombosis and haemorrhages, show a more irregular contour. Additionally, we show that increased plaque complexity parallels increased triglycerides-to-HDL ratio. Such ratio was shown to increase in metabolic syndrome and to mark insulin resistance.[32]. In turn, metabolic syndrome has been associated with vascular inflammation, a feature of plaque vulnerability.[33] Moreover, triglycerides-to-HDL ratio was observed to predict early vascular impairment in the young subjects [34] and to associate to extensive atherosclerotic disease,[35] indicating a strongly atherogenic role. Interestingly, total and LDL plasma cholesterol do not show a correlation with FD, and triglycerides only display a positive, but not statistically significant trend. This result in partially in contrast with previous literature suggesting that elevated plasma LDL increase plaque instability.[36] For this reason, integration of FD with other ultrasonographic markers of plaque instability may be of great interest, especially in view of the independence of FD from known risk features, such as degree of stenosis and echogenicity, as shown by our analyses.

### Study limitations

There are three main limitations in the use of fractal analysis. The outline of the carotid plaque involves a semi-automated algorithm, with a significant subjective contribution in the identification of plaque border, mainly in threshold adjustment. However, the degree of inter-observer agreement shows that this problem does not significantly alter the reproducibility of the technique. On the other hand, we could not assess the inter-observer variability for image acquisition, since only one operator (MM) examined the patients. Lastly, in the presence of calcification with posterior acoustic shadow this technique of little use, since plaque contour cannot be visualized. For this reason, in this study, we had to discard 4 subjects from the evaluation of gFD. While also in the analyzed subject some noise, which is intrinsic in every imaging modality, may have somewhat influenced the identification of plaque profile, B-mode ultrasound at the low tissue depth and high frequency employed has a high lateral resolution, and highly artefactual images, i.e. those in which the plaque border was hidden by an acoustic shadow were carefully excluded. Thus, we believe that the FD obtained are fairly accurate.

On the technical side, we were not able to analyze 3D image of the carotid artery due to software and hardware limitations at our institution. Further research in 3D imaging may enrich the data on plaque irregularity. We decided to perform fractal analysis on long axis views of the carotid instead of multiple transversal images of the artery, since the established prognostic value of plaque irregularity was indeed demonstrated on long axis projections.[3, 6]

The present study featured a small sample size in a cross-sectional evaluation, and could not explore the association with a relevant clinical outcome such as plaque rupture or cerebrovascular events. It may be considered a proof of principle work, establishing the feasibility and the reproducibility of fractal analysis in the evaluation of carotid plaques, and the association of mFD with an altered lipid profile. Further adequately powered studies are needed to establish fractal analysis as a risk factor and marker of carotid plaque instability and thus to validate the use of FD technique in the clinical assessment of carotid plaques. This will require prospective studies and histopathologic evaluation due to the lack of a gold standard for the assessment of carotid plaque irregularity.

## Conclusions

Fractal analysis is a novel, readily available and inexpensive technique for the quantitative measurement of plaque irregularity. The high inter-observer agreement obviates the subjectivity intrinsic in semi-automated delineation of the plaque border. The correlation between low HDL-C levels, high triglycerides-to-HDL ratio and plaque border mFD suggests a role for altered lipid profile in the acquisition of morphologic features of plaque instability. Further studies are needed to validate the use of fractal analysis in the risk assessment of carotid plaques.

## Supporting information

S1 Database Patients database. Study database, employed for the presented analysis.
(PDF)

S2 Database Reproducibility database. Results of the blinded analyses for reproducibility assessment.
(PDF)
