# Supplementary material for: Fractal analysis of plaque border, a novel method for the quantification of atherosclerotic plaque contour irregularity, is associated with pro-atherogenic plasma lipid profile in subjects with non-obstructive carotid stenoses
Source: PLoS One. 2018 Feb 12;13(2):e0192600. doi: 10.1371/journal.pone.0192600 (PMC5809053; doi:10.1371/journal.pone.0192600)
Supplement: S1 Database — Study database, employed for the presented analysis. (PDF) [file pone.0192600.s001.pdf]

ORDINE ARR	Sex	Age	BMI	GFR	FRS	FamHisCAD
46	1	58	19	80	9	0
64	1	68	25			1
30	1	64	29	54	8	1
24	1	82	30	63	18	1
3	2	77	27	51	9	0
6	2	64	20		1	1
36	2	63	23	104	3	0
12	1	73	21	79	13	0
26	2	63	26	65	2	0
29	2	69	22	49	4	0
5	2	69	24	56	3	0
14	2	49	21	77	1	1
8	2	71	20	61	1	0
9	1	64	26	95	12	0
33	2	54	38	107	3	1
10	1	71	25	71	14	0
13	1	67	29	92	14	1
22	2	65	30		5	0
2	2	74	22	53		0
15	2	74	25	44	7	1
37	2	76	18		8	0
7	2	74	28	81	5	0
35	1	79	17		23	0
17	1	77	24	78	22	0
28	2	68	27	78	6	0
41	1	79	25		22	0
40	1	73	27	72	20	1
25	1	68	28	111	17	0
19	1	59	28	107	9	0
27	1	75	27	64	25	0
39	1	73	28	85	17	0
34	1	67	28	82	9	1
63	1	77	23	58	25	0
38	1	75	25	69	28	0
18	1	67	26	68	18	0
23	2	77	19	43	7	0
11	1	59	28	123	9	1
4	1	77	26	62	20	1
16	2	77	28	35	11	0
50	2	83	33	51	20	0
1	1	76	24	68	24	0
20	1	84	27	47	23	0

FamHisSTROKE	HTN	Diabetes	Dyslipidemia	Smoking	PersHisCAD	ACS
0	0	0	0	2	0	0
0	1	0	0	0	0	0
0	1	0	0	2	0	0
0	1	0	0	2	0	0
1	1	0	0	0	0	1
0	0	0	1	0	0	0
0	0	0	1	0	0	0
0	0	0	1	1	0	0
0	0	0	1	1	0	0
1	1	0	1	0	0	0
0	1	0	1	0	0	0
0	1	0	1	0	0	0
0	1	0	1	0	0	0
0	1	0	1	1	0	0
0	1	0	1	1	0	0
0	1	0	1	2	0	0
0	1	0	1	2	0	0
1	1	0	1	2	0	0
0	1	0	1	2	0	0
0	1	0	1	2	0	0
0	1	0	1	2	0	0
0	1	0	1	0	0	0
0	0	1	0	0	0	0
1	1	0	0	0	0	0
0	1	1	0	0	0	0
0	1	0	0	1	0	0
0	1	0	0	2	0	0
0	0	1	1	2	0	0
0	0	1	1	2	0	0
0	1	0	1	0	0	0
0	1	0	1	0	1	0
0	1	1	1	0	0	0
0	1	0	1	1	0	0
0	1	0	1	2	1	0
0	1	0	1	2	1	1
0	1	0	1	2	1	0
0	1	1	1	2	0	0
0	1	1	1	2	0	0
0	1	1	1	2	1	1
1	1	0	1	0	0	0
0	1	0	1	2	0	0
0	1	0	1	2	1	1

glicemia	ACEi	Bblock	Statin	Diuretics	ARB	Antiplatelet
82	0	0	0	0	0	0
	1	0	0	1	0	1
91	0	0	0	1	1	0
95	0	1	0	0	1	1
90	1	1	1	1	0	0
88	0	0	1	0	0	1
93	0	0	0	0	0	0
	0	0	1	0	0	1
	0	0	1	0	0	1
100	0	0	1	0	1	1
75	1	0	1	0	0	0
83	0	0	1	0	1	1
82	0	0	1	0	0	0
86	1	1	1	0	0	1
94	0	1	1	0	1	1
131	1	0	1	0	0	1
144	0	0	1	0	1	1
	0	0	0	0	0	0
87	0	0	0	1	0	0
	0	1	1	0	1	1
	0	1	1	0	0	1
92	1	0	1	1	0	0
248	0	1	0	0	0	1
119	0	0	0	0	1	1
123	1	1	0	0	0	1
	0	0	0	1	1	1
108	0	1	0	1	0	1
170	0	0	0	0	0	1
124	0	0	1	0	0	1
94	0	0	1	0	1	1
129	1	1	1	0	0	0
98	0	0	1	0	1	0
88	1	0	0	0	0	1
	1	1	0	0	1	1
118	0	1	1	0	1	1
102	0	0	0	0	1	1
139	1	0	0	0	0	0
124	1	0	1	0	0	0
151	0	0	1	0	1	1
90	0	0	1	1	1	1
96	0	1	0	1	1	0
105	1	1	1	1	0	1

Iresistant HTN	Main plaque side (1=left, 2=right)	Max stenosis: <50; 2: 50-70	Ipx PSV	GSC (1=lipid-	C-IMT	CHOL TOT
0	1	1	64	1	0,76	183
0	2	1		2	0,75	
0	1	2	167	2	1,29	178
0	2	1	70	2	1,08	179
1	1	2	128	1	1,34	124
0	1	1	107	2	0,65	171
0	2	1	82	2	0,77	164
0	2	2	153	2	1,01	191
0	1	1	73	1	0,73	218
0	2	1	46	2	0,98	201
0	2	2	128	2	0,67	209
0	1	2	137	1	0,73	166
0	2	2	137	1	0,8	184
0	2	1	46,9	2	0,89	177
0	2	1		2	0,78	182
0	2	2		2	0,81	174
0	2	2	143	2	1,1	148
0	2	1	90	2	0,72	275
0	2	1	76	1	1,09	237
0	2	1	52	2	1,32	147
0	2	2	108	2	0,76	182
1	2	2	129	2	0,76	159
0	2	2	125	1	0,91	133
0	2	1	112	1	1,24	188
0	2	1		2	0,73	227
1	2	1		2	0,82	
1	1	1	92	2	0,84	199
0	1	1	45	1	0,88	185
0	2	2	142	1	0,64	171
0	1	1	80	2	0,85	158
0	1	2	132	2	1,11	144
0	1	2	140	2	0,82	171
0	1	2		1	0,82	
0	2	2		2	0,75	133
0	1	1	67	2	0,9	114
0	1	1	113	2	0,75	133
0	2	1	179	2	0,88	196
0	2	1	76	2	0,9	155
0	1	2	173	2	1,3	161
1	2	1	125	2	1,06	151
1	2	2	126	1	0,75	159
1	2	1	113	2	0,78	182

HDL-C	TRIG	APO AI	APOB	HS-CRP	LDL-C	ApoB/ApoA1
36	139	113	86	2,46	119,2	0,76
39	139	114	62	9,46	111,2	0,54
38	126	154	82	0,48	115,8	0,53
40	82				67,6	
55	52	165	78	1,21	105,6	0,47
36	100	114	61	0,74	108	0,54
38	130	169	85	0,74	127	0,50
41	127	162	92	4,75	151,6	0,57
56	161	174	72	0,93	112,8	0,41
46	151	191	97	1,13	132,8	0,51
52	46	167	59	0,6	104,8	0,35
61	99	200	73	0,54	103,2	0,37
32	106	162	88	1,11	123,8	0,54
47	151	147	78	5,57	104,8	0,53
42	118	182	64	0,48	108,4	0,35
36	64	147	57	0,48	99,2	0,39
67	154	173	143	2,21	177,2	0,83
73	146				134,8	
28	121	115	63	2,6	94,8	0,55
51	166	147	72	6,18	97,8	0,49
41	97	179	61	1,56	98,6	0,34
45	184	132	56	12,8	51,2	0,42
45	133	153	89	4,73	116,4	0,58
46	256	150	103	1,21	129,8	0,69
39	171	135	90	3,09	125,8	0,67
32	157	134	77	0,55	121,6	0,57
36	164	112	81	1,2	102,2	0,72
45	95	130	76	0,73	94	0,58
47	163	139	52	3,34	64,4	0,37
63	119	173	69	0,48	84,2	0,40
36	178	117	56	0,48	61,4	0,48
48	87	88	83	1,78	48,6	0,94
37	60	122	47	0,48	84	0,39
44	170	183	83	0,48	118	0,45
47	97	128	67	1,18	88,6	0,52
36	129	147	65	0,79	99,2	0,44
35	230	117	62	0,67	70	0,53
39	227	129	72	7,57	74,6	0,56
33	248	103	100	0,48	99,4	0,97

High CVD risk	mFD	Trig:HDL	gFD
0	1,1602	3,86111111	1,17555
0	1,1535		1,1394
0	1,1746	3,56410256	1,1638
0	1,1866	3,31578947	1,1986
0	1,1336	2,05	1,1509
0	1,1071	0,94545455	1,0997
0	1,1404	2,77777778	1,1623
0	1,1460	3,42105263	1,1354
0	1,0119	3,09756098	1,0981
0	1,1066	2,875	1,0901
0	1,1981	3,2826087	1,16125
0	1,1057	0,88461538	
0	1,1175	1,62295082	1,15195
0	1,1712	3,3125	1,18785
0	1,1222	3,21276596	1,08925
0	1,1430	2,80952381	1,1289
0	1,0909	1,77777778	1,131
0	1,1363	2,29850746	1,13335
0	1,0995	2	1,1035
0	1,1473	4,32142857	1,16255
0	1,1617	3,25490196	1,16575
0	1,1564	2,36585366	1,1211
1	1,1447	4,08888889	1,16635
1	1,2207	2,95555556	1,1713
1	1,1268	5,56521739	1,1598
1	1,1345		
1	1,1065	4,38461538	
1	1,1124	4,90625	1,1274
1	1,1669	4,55555556	1,1458
1	1,1617	2,11111111	1,17385
1	1,1667	3,46808511	1,1438
1	1,0517	1,88888889	1,12065
1	1,1087		1,13715
1	1,1336	4,94444444	1,11555
1	1,1059	1,8125	1,1069
1	1,0810	1,62162162	1,06415
1	1,1171	3,86363636	1,1325
1	1,1145	2,06382979	
1	1,1992	3,58333333	1,2143
1	1,1661	6,57142857	1,16495
1	1,1408	5,82051282	1,1194
1	1,1670	7,51515152	1,27865
