# Supplementary material for: Fractal analysis of plaque border, a novel method for the quantification of atherosclerotic plaque contour irregularity, is associated with pro-atherogenic plasma lipid profile in subjects with non-obstructive carotid stenoses
Source: PLoS One. 2018 Feb 12;13(2):e0192600. doi: 10.1371/journal.pone.0192600 (PMC5809053; doi:10.1371/journal.pone.0192600)
Supplement: S2 Database — Results of the blinded analyses for reproducibility assessment. (PDF) [file pone.0192600.s002.pdf]

ID patient	ID Image	FD-Op1	FD-Op2
63	63.2	1,1656	1,1662
2	2.1	1,1075	1,1162
64	64.1	1,1253	1,1414
29	29.1	1,0736	1,0725
50	50.1	1,1638	1,2023
3	3.2	1,1682	1,1538
26	26.2	1,1843	1,1737
33	33.1	1,0563	1,0547
38	38.1	1,0975	1,1083
22	22.1	1,1304	1,133
17	17.1	1,1219	1,1236
46	46.2	1,1909	1,2003
28	28.1	1,1928	1,1821
13	13.1	1,1711	1,1772
34	34.2	1,1896	1,1885
27	27.2	1,186	1,1817
16	16.2	1,2294	
30	30.2	1,153	1,1565
8	8.1	1,1864	1,1832
35	35.1	1,188	1,1859
